# Obesity predicts mortality stronger in adult‐onset asthma than in age‐ and sex‐matched controls

**DOI:** 10.1002/clt2.70011

**Published:** 2024-11-28

**Authors:** Helena Backman, Caroline Stridsman, Anne Lindberg, Eva Rönmark, Linnea Hedman

**Affiliations:** ^1^ Department of Public Health and Clinical Medicine Umeå University Umea Sweden

**Keywords:** epidemiology, obstructive airways disease, prognosis, risk factors

To the Editor,

Several studies have shown that being obese is associated with an increased risk of developing asthma, especially adult‐onset asthma, as well as more severe asthma symptoms.^1^, ^2^ Obesity can affect the respiratory system in several ways. Excess body fat can mechanically lead to a decrease in lung volume, which can make breathing more difficult. Obesity is also associated with a state of chronic low‐grade inflammation,^3^ which can contribute to the development of asthma and exacerbate asthma symptoms.^1^ In addition, obesity can lead to changes in the structure and function of the airways that make them more susceptible to inflammation and cause obstruction.^1^, ^4^ Obesity associates with mortality both in the general population and among adults with asthma,^1^, ^3^, ^5^ but most studies in adults are done by stratifying population‐samples by presence and absence of asthma and thus with often slightly different age and more women in those with asthma.^6^ Less is known about whether the obesity‐mortality association is stronger in adult‐onset asthma than in adults without asthma when taking age and sex into account.

In this hypothesis‐generating study, we aimed to explore the association between obesity and mortality in patients with adult‐onset asthma compared to age‐ and sex‐matched controls.

## 1 METHODS

During 1995–1999, 309 adults (19–61 years, 65% women) with newly onset asthma were identified in primary care and referred to the Obstructive Lung Disease in Northern Sweden (OLIN) Studies where a diagnosis of asthma and bronchial variability was confirmed.^2^ *N* = 309 sex‐ and age‐matched controls without asthma were also included. Body mass index (BMI, kg/m^2^) at baseline was categorized into normal weight (BMI 20–24.9), underweight (BMI < 20), overweight (BMI 25–29.9) and obesity (BMI ≥ 30). Based on the unique Swedish personal identity numbers, mortality data was linked until November 2023. Person‐years were calculated as the number of years from baseline examination to death or November 2023, whichever occurred first. Means were compared across groups using *T*‐test or ANOVA, while the Chi‐squared test was used to compare proportions, as appropriate. Statistical significance was set at *p* < 0.05. Cox proportional hazards regression was used to calculate hazard ratios (HR) for BMI categories (normal weight as reference) adjusted for smoking habits, age and sex, separately among cases and controls.

## 2 RESULTS

**FIGURE 1:**
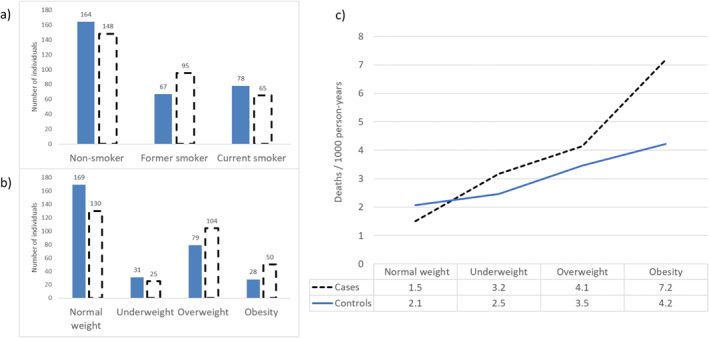
(A) Smoking habits at baseline, (B) body mass index (BMI) categories at baseline, and (C) number of deaths by 1000 person‐years during follow‐up by baseline BMI category, for 309 cases with incident adult‐onset asthma (black dashed) and 309 age‐ and sex‐matched controls (blue) without asthma at baseline.

The mean age at baseline was 37 years, and there were 48% non‐smokers, 31% former smokers, and 21% current smokers among the cases, compared to 53%, 22% and 25% in controls. There were more individuals with obesity in cases versus in controls (16% vs. 9%, *p* < 0.001) (Figure 1A,B). The cumulative mortality was *n* = 27 (9%) in cases and *n* = 21 (7%) in the controls.

The number of deaths/1000 person‐years during follow‐up are illustrated by BMI category in Figure 1C. Among cases, the HR (95% CI) was 3.6 (0.7–19.2) for underweight, 1.7 (0.6–5.2) for overweight, and 3.5 (1.1–11.7) for obesity. The corresponding figures for controls were 1.0 (0.2–4.8), 0.9 (0.3–2.6) and 1.2 (0.3–4.4).

## 3 DISCUSSION

Obesity is increasing in many parts of the world and hence, it's burden in terms of morbidity and mortality is also expected to increase. Asthma has also been on the increase in some parts of the world, especially in areas with rapid urbanization and those dominated by western lifestyle.^1^ Given that obesity may not only cause but also exacerbate asthma, a poorer prognosis for obese individuals with asthma is also a logical deduction. However, asthma is rarely the main cause of death in westernized societies today, and thus the explanation for our finding of a higher all‐cause mortality due to obesity in adult‐onset asthma than in adults without asthma is probably multifaceted.

Adult‐onset asthma is more often a non‐allergic and non‐eosinophilic asthma phenotype as compared to childhood asthma, and it is also less likely to remit^7^ or respond well to inhaled corticosteroid treatment, which is the cornerstone in asthma treatment.^1^ Beside obesity, adult‐onset asthma is further associated with other comorbidities such as type‐2 diabetes, metabolic syndrome, cardiovascular disease and depression.^8^ Adult‐onset asthma is also associated with increased levels of blood neutrophils,^4^ and there may be an interplay between poor asthma control, inflammation and obesity.^9^ Thus, beside the purely mechanic effect of obesity on the lungs and airways, co‐occurrence with other conditions along with increased levels of different adipokines and systemic inflammation may partly explain the obesity‐mortality finding.

Potential contributors to the stronger obesity‐mortality association in adult‐onset asthma include socioeconomic, environmental and lifestyle factors associated with both asthma, obesity and other diseases. Regarding asthma, a lower level of education may affect both asthma control and prognosis.^6^ Smoking, harmful occupational exposures, lower levels of physical activity, and weight gain are more common in individuals with lower socioeconomic status, and may cause both obesity and asthma or interact with asthma with regards to development of airway obstruction.^1^ Thus, multiple risk factors, along with development of other lifestyle‐related diseases, may be of importance for the complex mechanisms impacting prognosis of adult‐onset asthma.

Strengths of the current study include the careful clinical examinations at baseline assuring cases as having recent onset asthma in adulthood, confirming presence of bronchial variability and asthma diagnosis.^2^ Weaknesses include the limited sample size and few events, as well as the lack of analyses on comorbidities, specific causes of death and change in BMI during follow‐up.

In conclusion, our Swedish study shows that obesity predicts mortality stronger in adult‐onset asthma than in age‐ and sex‐matched controls, highlighting the need for phenotyping, personalized medicine and regular follow‐ups. The reasons for this increased obesity‐mortality association in adult‐onset asthma are probably several and multifaceted, however, prevention and treatment of obesity is important.

## AUTHOR CONTRIBUTIONS

**Helena Backman**: Conceptualization; writing—original draft; methodology; writing—review & editing; formal analysis; funding acquisition. **Caroline Stridsman**: Conceptualization; writing—review & editing; methodology. **Anne Lindberg**: Conceptualization; investigation; writing—review & editing; methodology. **Eva Rönmark**: Funding acquisition; writing—review & editing; investigation; conceptualization; methodology; project administration. **Linnea Hedman**: Conceptualization; writing—review & editing; methodology.

## CONFLICT OF INTEREST STATEMENT

HB, ER and LH have no conflicts of interest to declare. AL reports personal fees for lectures at educational events outside the submitted work from Boehringer Ingelheim and AstraZeneca, and personal fees for Advisory Board at AstraZeneca, Boehringer Ingelheim, GlaxoSmithKline, and Novartis. CS reports personal fees outside the submitted work for lectures at educational events from Boehringer Ingelheim, Novartis and AstraZeneca, and fees for manuscript writing, outside the submitted work, from Chiesi and TEVA.

## Data Availability

The data that support the findings of this study are available from the corresponding author upon reasonable request.
